# Development of a Wireless Displacement Measurement System Using Acceleration Responses

**DOI:** 10.3390/s130708377

**Published:** 2013-07-01

**Authors:** Jong-Woong Park, Sung-Han Sim, Hyung-Jo Jung, Billie F. Spencer

**Affiliations:** 1 Department of Civil and Environmental Engineering, KAIST, Daejeon 305-701, Korea; E-Mails: jwp@kaist.ac.kr (J.-W.P.); hjung@kaist.ac.kr (H.-J.J.); 2 School of Urban and Environmental Engineering, Ulsan National Institute of Science and Technology (UNIST), Ulsan 689-798, Korea; 3 Department of Civil and Environmental Engineering, University of Illinois at Urbana-Champaign, Champaign, IL 61801, USA; E-Mail: bfs@illinois.edu

**Keywords:** displacement estimation, bridge displacement, acceleration, displacement, wireless smart sensor network, structural health monitoring

## Abstract

Displacement measurements are useful information for various engineering applications such as structural health monitoring (SHM), earthquake engineering and system identification. Most existing displacement measurement methods are costly, labor-intensive, and have difficulties particularly when applying to full-scale civil structures because the methods require stationary reference points. Indirect estimation methods converting acceleration to displacement can be a good alternative as acceleration transducers are generally cost-effective, easy to install, and have low noise. However, the application of acceleration-based methods to full-scale civil structures such as long span bridges is challenging due to the need to install cables to connect the sensors to a base station. This article proposes a low-cost wireless displacement measurement system using acceleration. Developed with smart sensors that are low-cost, wireless, and capable of on-board computation, the wireless displacement measurement system has significant potential to impact many applications that need displacement information at multiple locations of a structure. The system implements an FIR-filter type displacement estimation algorithm that can remove low frequency drifts typically caused by numerical integration of discrete acceleration signals. To verify the accuracy and feasibility of the proposed system, laboratory tests are carried out using a shaking table and on a three storey shear building model, experimentally confirming the effectiveness of the proposed system.

## 1. Introduction

Displacement measurements provide useful information for various engineering applications such as structural health monitoring (SHM), earthquake engineering, and system identification. Displacement responses from a structure can be obtained either by: (1) contact or (2) non-contact type sensors (see Table 1). The linear variable differential transformer (LVDT), one of the most commonly used contact type devices, requires a connection link between the structure and the fixed reference point; applications to large civil structures such as bridge deflection measurements are quite challenging due to the difficulties in sensor installation. GPS can be an attractive alternative as displacement can be conveniently measured, but the use of GPS is limited to long-period, large deflection structures such as high-rise structures and long-span bridges due to its relatively low sampling rate and accuracy [1–3].

Non-contact type methods that can remotely measure displacement include laser Doppler vibrometers (LDVs) [12,13], total station [15], and vision-based systems [16–18]. While displacement is conveniently measured, the non-contact type approaches can have accuracy issues due to the mounting condition of the devices. In particular, large civil structures such as long-span bridges are typically constructed over seas, rivers, or roads where the sensor installation is difficult; finding appropriate mounting positions is challenging.

The difficulties found in the contact and non-contact type approaches can be resolved by introducing reference-free, indirect estimation approaches for displacement acquisition. Measurements such as acceleration [7,8], velocity, and strain [6,9–11] are typically employed as they can be obtained without reference points. In particular, accelerometers are commonly used in dynamic testing of structures due to their installation convenience and relatively low cost and noise; the indirect estimation using acceleration responses has great potential to be widely adopted for obtaining displacement information of structures.

The displacement estimation using acceleration measurements is based on the fact that the double integration of acceleration is equal to displacement. However, the numerical integration requires the initial conditions of the displacement to be determined, which are generally unavailable. In addition, the double integration involves intrinsic errors due to measurement noise and imperfect information in measured discrete acceleration signals, typically resulting in the low frequency drift in the estimated displacement. Thus, minimizing the errors caused by the numerical double integration is the most important challenge for any acceleration-based approach [19].

Park *et al.* [8] suggested initial velocity estimation methods for directly integrating acceleration to determine displacement. The method is independent of initial conditions and performs integration by dividing acceleration data into several segments to improve accuracy. However, as the size of the segments is empirically determined, implementing the automated calculation on a smart sensor is difficult. In addition, a large number of trial-and-error iterations need to be performed to find the initial conditions. Thus, this approach is considered inappropriate for smart sensor applications while accurately calculating displacement. Kandula *et al.* [20] proposed a signal model for acceleration as a sum of exponentially damped sinusoidal signals. The noise-free acceleration can be modeled and then twice integrated to obtain a displacement response. However, this approach involves a matrix inverse of relatively large datasets, which can cause long calculation times and possible instability in the smart sensor. Also, the acceleration data is processed in separate blocks for non-stationary processes without a consistent guideline, which hinders automated processing. Lee *et al.* [7] developed a dynamic displacement estimation method using acceleration to remove the drift error by filtering out low frequency components. Defined as a boundary value problem, this approach only requires prior information regarding the lowest frequency, while the initial condition of displacement is unnecessary. While limited to zero-mean displacements, this approach is seen to be promising because it is computationally efficient, cost-effective, reference-free, and accurate for zero-mean displacements. However, accelerometers need complex wiring from the desired measurement place to a central base station, in which measured signals can be contaminated by electric noise.

The applicability of the acceleration-based displacement estimation can be greatly enhanced by integrating with smart sensors to provide full displacement information about large civil structures. Smart sensors have been recognized as a new paradigm in civil infrastructure monitoring, being expected to overcome limitations that have hindered widespread adoption of traditional wired monitoring systems [21]. Key features of the smart sensor are intelligent on-board computing, wireless communication, cost effectiveness, and sensing capability [22], which enable dense networks of sensors essential for reliable and accurate assessment of structural health in full-scale structures. With the acceleration-based approaches embedded on smart sensors, a network of smart sensors can utilize the computing power to perform distributed in-network data processing, converting measured acceleration signals to displacement at every sensor location. Thus, detailed displacement information of a structure can be obtained from densely deployed smart sensors.

This paper presents a wireless displacement measurement system based on acceleration responses. The proposed system is designed to perform the distributed in-network data processing to transform measured accelerations to dynamic displacements at each sensor location. Due to limited resources available in the smart sensor such as the battery power and the computational capability, the embedded processing for displacement estimation is optimized to minimize the power consumption during the computation while maximizing the stability of the system. The validity of the proposed system is experimentally demonstrated on a shaking table test with various frequency responses and on a three storey shear building model subjected to a random excitation.

## 2. Displacement Estimation Using Acceleration

A wide variety of engineering applications employ acceleration to identify desired information because acceleration sensors are generally cost-effective, convenient to install, and have relatively low noise. Compared to the displacement sensors that require fixed reference points, the installation convenience of the accelerometer sensors has great advantages in civil engineering applications in which the reference points are often unavailable. Thus, acceleration is considered to be appropriate for indirectly obtaining displacement responses of civil engineering structures.

We investigated acceleration-based displacement estimation algorithms based on the following criteria for possible use with the smart sensor:

- Low frequency errors can be removed.
- Initial conditions are not required in the calculation.
- Automated calculation.
- Computational efficiency.

Knowing that the smart sensors have limited resources and should be operated autonomously, the criteria of the computational efficiency and the automated calculation are included. As previously reviewed, the approach proposed by Lee *et al.* [7] is seen to be suitable for the wireless displacement estimation system, satisfying the criteria with distinguished computational efficiency and stability.

For completeness, the displacement estimation approach is briefly described. Consider the following optimization problem to determine displacement from measured acceleration:

(1) $$\underset{u}{\mathrm{Min}}\Pi =\frac{1}{2}{\left\|{\text{L}}_{a}({\text{L}}_{c}\text{u}-{\left(\Delta t\right)}^{2}\bar{\text{a}})\right\|}_{2}^{2}+\frac{\lambda^{2}}{2}{\left\|\text{u}\right\|}_{2}^{2}$$

where **u** , Δ*t*, and **ā** are the estimated displacement, sampling time, and measured acceleration, respectively. **L***_a_* is the integration operator of the discretized trapezoidal rule, **L***_c_* is the second-order differential operator, ‖·‖_2_ denotes 2-norm of a vector, and *λ* represents the optimal regularization factor that adjust the contribution of the second term in the minimization problem. The optimal solution to this problem is the displacement that is close to the numerical double integration of the measured acceleration by the first term in Equation (1), while the magnitude is kept from becoming large due to the drift problem by having the second term. The solution to Equation (1) can be written as:

(2) $$\text{u}={\left({\text{L}}^{T}\text{L}+\lambda^{2}\text{I}\right)}^{-1}{\text{L}}^{T}{\text{L}}_{a}\bar{\text{a}}{\left(\Delta t\right)}^{2}=\text{C}\bar{\text{a}}{\left(\Delta t\right)}^{2}$$

where **L** = **L***_c_***L***_a_* and the superscript *T* denotes the matrix transpose. Note that this approach assumes the displacement is a zero-mean process.

**Figure 1.**
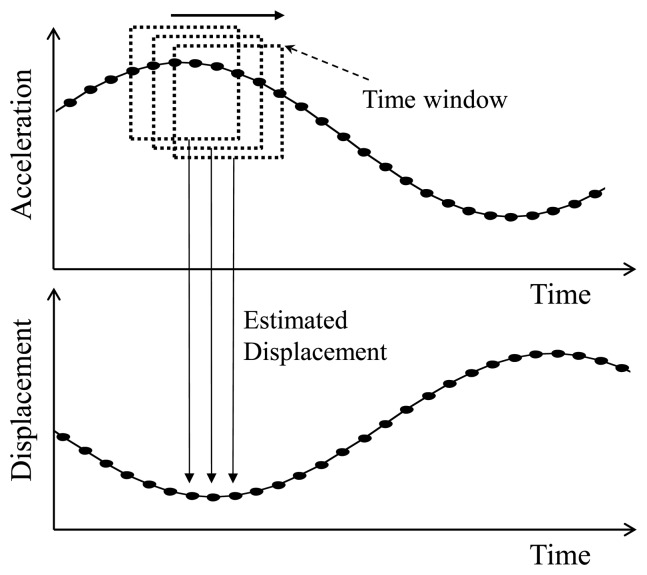
Displacement reconstruction scheme using overlapping time windows.

Because boundary conditions at the beginning and end of the displacement in the time domain are unknown, the displacement calculated using Equation (2) has significant errors near the boundaries. This problem can be resolved by using an overlapping moving window approach, which is based on the fact that the displacement estimation is accurate at the center, while the error increases near the boundaries. The moving windows are selected as shown in Figure 1, each of which is used to calculate the displacement using Equation (2). The displacement values at the center of each window are collected to obtain the full displacement time history. This process is equivalent to applying an FIR filter of ***C*** Δ*t*^2^ to the measured acceleration **ā**. In addition, the optimal regularization factor can be determined as:

$$\lambda_{\text{optimal}}=46.81N_{d}^{-1.95}.$$

## 3. Wireless Displacement Measurement System

Combined with the displacement estimation algorithm, the smart sensor enables the wireless displacement measurement system with enhanced applicability to full-scale civil structures. The wireless communication associated with the smart sensor network removes the cabling between sensors and the central data repository that needs significant effort and cost as in the wired sensor systems, and thus allows a dense network of sensors to be deployed in large structures. Each sensor node in the network can conduct sensing and distributed computing to convert measured acceleration to displacement, providing full structural displacement information.

**Figure 2.**
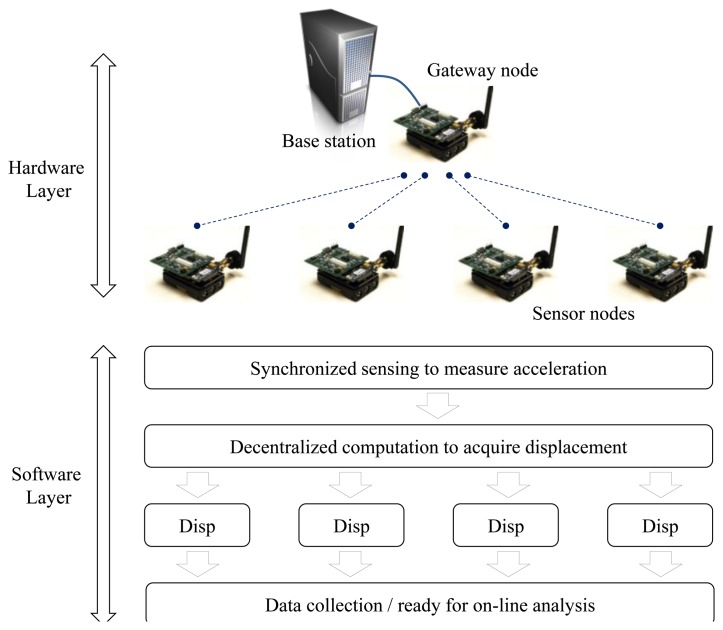
Schematic view of the wireless displacement measurement system.

The proposed wireless displacement measurement system is based on the combination of the smart sensors and the displacement estimation algorithm to provide a practical means of acquiring the detailed displacement profile of full-scale civil structures. The system consists of: (1) hardware layer including a base station, a gateway node and smart sensor nodes, and (2) software layer embedded in each sensor node for data acquisition, displacement estimation, and network operation (see Figure 2). The base station is to interface with the network, the gateway node controls the network, and the sensor node is for sensing, computation, and communication. With the sensor hardware deployed on a structure, the smart sensor network measures acceleration responses and produces dynamic displacement through the software layer. As each sensor node uses its own measured acceleration for the calculation, the software layer that implements the displacement estimation employs the distributed independent processing scheme in which each sensor node process measured data without information sharing with neighbor nodes [23].

The proposed system inherits all features and limitations of the smart sensor and the displacement estimation algorithm on which the system is based. For example, operational capabilities such as wireless communication range, computation time, and maximum data length depend on mostly smart sensor platforms. In addition, the proposed system is limited to zero-mean displacements as the selected FIR-filter type displacement estimation algorithm is.

To realize a reliable wireless displacement measurement system, both hardware and software layers of the system are carefully considered with: (1) the selected sensor hardware that consists of MEMSIC's Imote2 smart sensor platform and ISM400 sensor board and (2) the implementation of the displacement estimation approach based on the distributed independent processing.

### 3.1. Imote2 Smart Sensor Platform

**Figure 3.**
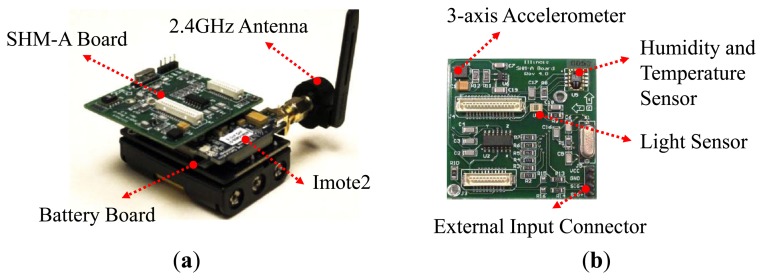
Hardware components of smart sensor node: (**a**) Imote2; (**b**) ISM400 sensor board.

For the hardware layer of the wireless displacement measurement system, MEMSIC's Imote2 sensor platform shown in Figure 3 is selected due to its reliability, powerful computing capability, and sufficient memory spaces. Imote2 is a high-performance wireless, computing module with Intel's PXA271 XScale^®^ processor running at 13–416 MHz with memory spaces of 256 kB SRAM, 32 MB FLASH, and 32 MB SDRAM. The powerful processor and large memory spaces enable long-term measurement as well as on-board processing of large data. Imote2 uses 2.4 GHz wireless communication with either the on-board or external antenna.

As shown in Figure 3, the Imote2 interfaces with sensor boards that can measure data such as acceleration, strain, temperature, humidity, and light, depending on the attached sensor boards. As acceleration is required in this study, MEMSIC's ISM400 sensor board also known as SHM-A developed at the University of Illinois at Urbana-Champaign [24] is used. The ISM400 sensor board features a 3-axis accelerometer (ST Microelectronic's LIS344ALH as in Table 2 and an embedded Quickfilter QF4A512 that has a 4-channel, 16-bit analog to digital converter (ADC) and a signal conditioner with user-selectable sampling rates and programmable digital filters. The ISM400 sensor board also has temperature, humidity, and light sensors.

### 3.2. Implementation of the Displacement Estimation Algorithm on Imote2

The displacement estimation on the Imote2-based sensor network is implemented using the Illinois SHM Project (ISHMP) Services Toolsuite. SHM applications running on smart sensors require complex programming for essential components such as network-wide synchronized sensing, reliable wireless communication, networking between nodes, and algorithm implementations; developing a smart sensor application from scratch is challenging and time consuming. The ISHMP Service Toolsuite provides open-source middleware services implementing these components that can be used as building blocks to develop a new smart sensor application, significantly reducing time and effort in programming. More detailed information regarding the ISHMP Services Toolsuite can be found in Rice *et al.* [25].

The application, *Independent processing-based Displacement Estimation using Acceleration (IDEA)*, is developed to estimate dynamic displacement from measured acceleration data based on the distributed independent processing. The implementation of *IDEA* combines the displacement estimation algorithm with essential services such as network-wide sensing and wireless communication provided by the ISHMP Services Toolsuite. The services used to develop *IDEA* include:

- *Time Synchronization* to synchronize local clocks in each sensor node.
- *Unified Sensing* for measuring acceleration.
- *SensingUnit* to perform network-wide sensing utilizing *Time Synchronization* and *Unified Sensing* services.
- *ReliableComm* for reliable wireless communication.
- *RemoteCommand* for gateway and sensor nodes to interact with each other in a way that command messages are conveyed to sensor nodes that perform designated tasks such as sensing, computing, and sending data.

**Figure 4.**
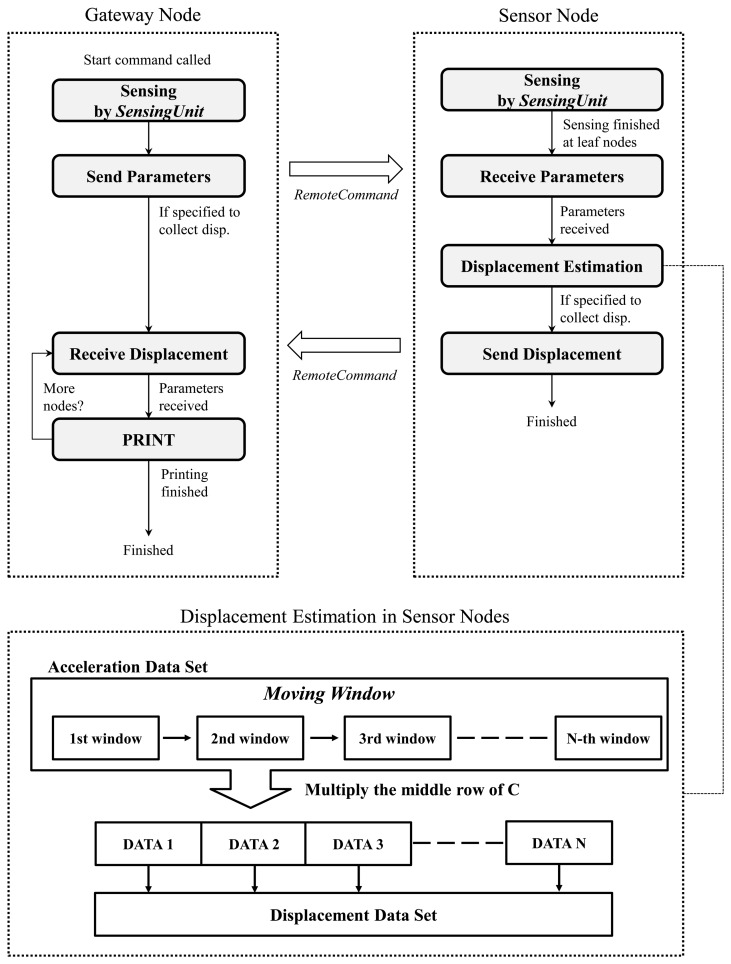
Flowchart of *IDEA*.

Note that the italicized denotes service names in the ISHMP Services Toolsuite. Due to these services, SHM applications for smart sensors can be more systematically and reliably developed. *IDEA* is developed by implementing the displacement estimation algorithm organized with the services as shown in the flowchart in Figure 4. Note that the lowest frequency of interest should be determined once by measuring acceleration before running *IDEA*. The network operation of the gateway and sensor nodes are shown on the left and right side of the Figure 4, respectively. When the sensor nodes finish sensing, the gateway node delivers parameters *(i.e.*, target frequency of the structure, sampling rate, and window size) that are required for calculating displacement. After the parameters are transferred to the sensor nodes, matrix **C** in Equation (2) is calculated; only the middle row of **C** is stored and used in the calculation of Equation (2) because the middle point of each moving window is selected as the corresponding displacement value as illustrated in Figure 1. This process is to reduce redundant use of memory spaces and computational power by a factor of the size of the time window, which is otherwise more than Imote2 can handle. Each moving window of the measured acceleration signal produces a point estimate of displacement, which is stored in the memory space of Imote2 as illustrated in the bottom of Figure 4.

When the displacement estimation process is finished, the gateway node can wirelessly receive the estimated displacement from each sensor node if the displacement is requested to the base station by users. Received from all sensor nodes, the estimated displacement in the gateway node is transferred to the base station. Note that the estimated displacements are collected at the base station for the verification purpose, which requires as many data packets as the centralized approach wirelessly transfers. *IDEA* can retain the displacements at each sensor node for further analysis to assess structural health, reducing the operation time and power consumption associated with the wireless communication.

## 4. Experimental Validation

To verify the performance of the implementation, two types of experiments are carried out: displacement estimation of (1) harmonic motions and (2) a three degrees-of-freedom structure under a random excitation.

### 4.1. Harmonic Motion Testing

**Figure 5.**
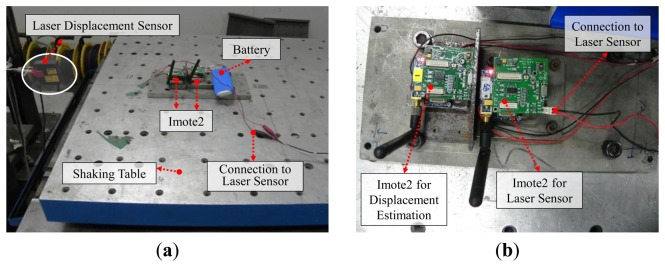
Experimental setup for harmonic motion testing: (**a**) overview of the validation test; (**b**) details of sensor arrangement.

The Imote2 sensor node is used to estimate displacement of a shaking table in harmonic motion. Two Imote2 sensor nodes and one laser displacement sensor are prepared on the shaking table (see Figure 5). The Imote2 sensor node on the left side in Figure 5(b) is for displacement estimation while the other Imote2 sensor node is for data acquisition from the laser displacement sensor through the external input channel on the ISM400 sensor board. Two Imote2 sensor nodes collect synchronized data so that the estimated displacements can be readily compared. Amplitudes of the harmonic motions are 2 mm, 4 mm, and 6 mm, each of which has four different frequencies of 0.5 Hz, 1 Hz, 1.5 Hz and 2 Hz. The size of the time window shown in Figure 1 is determined to be 2.68 times of the target periods (*i.e.*, inverse of the driving frequencies) as recommend by Lee *et al.* [7].

**Figure 6.**
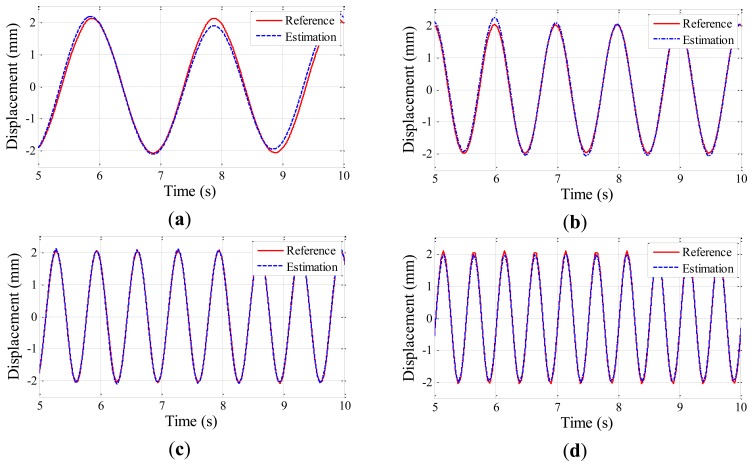
Excitation amplitudes of 2 mm: (**a**) 0.5 Hz excitation; (**b**) 1 Hz excitation; (**c**) 1.5 Hz excitation; (**d**) 2 Hz excitation.

**Figure 7.**
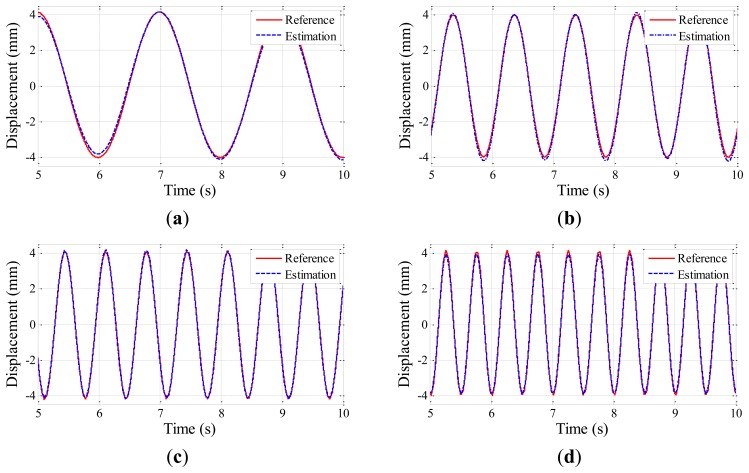
Excitation amplitudes of 4 mm: (**a**) 0.5 Hz excitation; (**b**) 1 Hz excitation; (**c**) 1.5 Hz excitation; (**d**) 2 Hz excitation.

**Figure 8.**
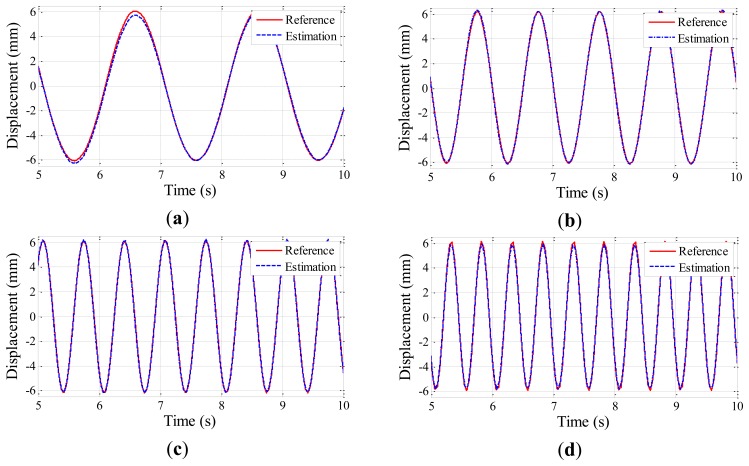
Excitation amplitudes of 6 mm: (**a**) 0.5 Hz excitation; (**b**) 1 Hz excitation; (**c**) 1.5 Hz excitation; (**d**) 2 Hz excitation.

The experiment is conducted five times for each testing case to obtain displacement data from the Imote2 and laser sensors. Time histories of the estimated displacements are shown in Figures 6, 7 and 8 for the amplitudes of 2 mm, 4 mm, and 6 mm, respectively; the estimated displacements are in good agreement with the reference. A difference measure in Equation (3) is introduced to quantitatively evaluate the estimation:

(3) $$d(u_{\text{est}},u_{\text{ref}})=\frac{{\left\|u_{\text{est}}-u_{\text{ref}}\right\|}_{2}}{{\left\|u_{\text{ref}}\right\|}_{2}}$$

where ‖•‖_2_ is a vector norm, and *u_est_* and *u_ref_* are the displacement time history data from the Imote2 and the laser sensor, respectively. The difference measure calculated for each case is shown in Table 3, ranging from 0.025 to 0.114. The difference is observed to decrease as the amplitude and frequency of the harmonic motion increase due to the intrinsic low frequency noise in the accelerometer of Imote2. As the overall difference levels shown in Table 3 can be considered reasonably small, the Imote2-based approach is shown to reliably produce the displacement of the harmonic motions.

### 4.2. Shear-Building Model Test

**Figure 9.**
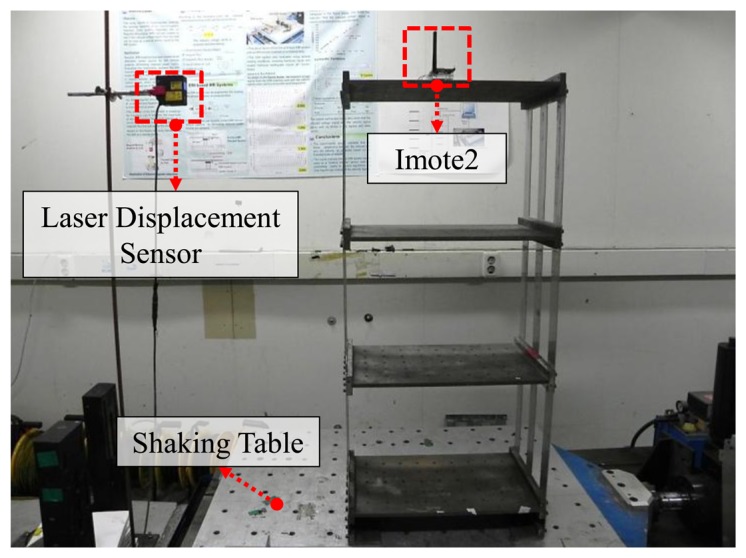
Experimental setup for shear building testing.

The wireless displacement measurement system is further validated using the 3-DOF shear building model on the shaking table as shown in Figure 9. The properties of the test structure are shown in Table 4. An Imote2 sensor node is installed on the top of the structure to estimate displacement at the location; a laser displacement sensor is also used to provide reference data (see Figure 9). The structure is excited with a random ground vibration having the amplitude of 0.1 mm and the bandwidth of 0–10 Hz.

Prior to the experiment, the acceleration data is collected to determine the size of the time window (see Figure 1). With the power spectrum of the measured acceleration, three natural frequencies of the structure are identified as 1.98 Hz, 5.35 Hz, and 8.11 Hz. Thus, the size of the time window is selected to be 2.68/1.98 Hz ≈ 1.35 s as calculated in the harmonic motion testing.

**Figure 10.**
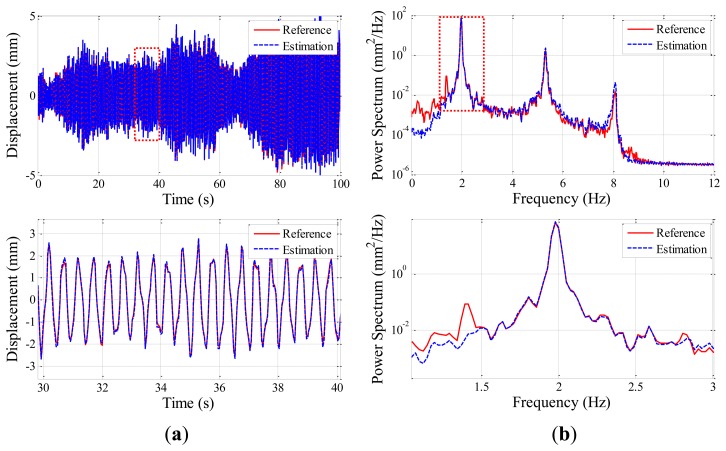
Comparison of the estimated and reference displacements: (**a**) time history; (**b**) power spectrum.

Displacement estimation using the Imote2 is performed with the sampling rate of 25 Hz, obtaining 2,500 data points. Figure 10 compares the estimated and reference displacements both in time and frequency domains, showing that the accurate estimation of the displacement by Imote2. To evaluate the accuracy in the time domain, the difference measure in Equation (3) is calculated to be 0.062. Considering the lowest frequency and standard deviation of the estimated displacement are 1.98 Hz and 1.9637 mm, respectively, the difference measure of 0.062 is greater than 0.034 when the frequency is 2 Hz and the amplitude is 2 mm in Table 3 as summarized in Table 5. The larger difference in the shear building test is due to the nature of the displacement estimation method employed in this study: the FIR filtering assumes the zero-mean process and thus frequency components lower than the target frequency (*i.e.*, 1.98 Hz in this test) may not fully contribute in the displacement estimation. As a result, the power spectrum of the estimated displacement has errors in the low frequency region as shown in Figure 10(a). Note that the effect of this low frequency error is small in the harmonic motion tests as most energy is concentrated on the driving frequency. Based on the observation both in the frequency and time domains, the Imote2-based WSSN can be considered to produce reasonably accurate displacement estimation.

## 5. Conclusions

This study proposes a low-cost wireless displacement measurement system based on acceleration responses, designed to acquire detailed displacement information about civil engineering structures. Among displacement estimation schemes using acceleration responses, the FIR filter-based method was selected due to its computational efficiency and stability. The displacement estimation algorithm was embedded on the Imote2 smart sensor platform with the middleware services of time synchronization, network-wide sensing, and reliable data communication, provided by the ISHMP Services Toolsuite. The Imote2-based displacement measurement system performs embedded data processing to estimate displacements at each sensor location using measured acceleration data. The validity of the proposed method was experimentally demonstrated in two laboratory-scale experiments. Harmonic motion testing was successfully carried out with the various frequencies and amplitudes, resulting in the accurate displacement estimation. To further validate the performance of the Imote2-based displacement measurement system for use in a structure with multiple natural frequencies, the random vibration test was conducted on the three-storey shear building model. The estimated displacement showed good agreement with the reference displacements measured from the laser displacement sensor. The series of displacement estimation tests described in this study showed substantial potential for the proposed approach to be used to provide detailed displacement information in large civil structures.

## Figures and Tables

**Table 1.** Displacement measurement methods.

**Type**	**Method**	**Reference**	**Resolution**
Contact type	LVDT	Reference-based	∼1 μm [4]
	GPS [1–3]	Reference-free	∼10 m [3]
		Reference-based	10∼20 mm [5]
	Indirect estimation [6–11] (acceleration, velocity, strain)	Reference-free	Device dependent 1)

Non-contact type	LDV [12,13]	Reference-based 2)	∼1 *μm* [14]
	Total Station [15]	Reference-based	∼1 mm at 200 m [15]
	Vision-based System [16–18]	Reference-based	∼0.15 mm at 70 m [18]

1) The resolution for each method is dependent on the performance of data acquisition devices.

2) The device location is considered to be a reference point because the measurement point is relative to it.

**Table 2.** LIS344ALH accelerometer specification [24].

**Parameter**	**Value**
Axes	3
Measurement range	±2 g
Noise density	50 μg/√Hz
Resolution	0.66 V/g
Power supply	2.4 V to 3.6 V
Temperature range	−40 to 85 °C
Supply current	0.85 mA

**Table 3.** Results of the validation test.

**Test Frequency (Hz)**	**Error (%)**		

	**2 mm**	**4 mm**	**6 mm**
0.5	11.4	6.7	4.1
1	5.6	4.6	2.5
1.5	4.5	3.6	2.3
2	3.4	2.7	2.5

**Table 4.** Structural parameters of the model.

**Parameters**	**Value**
Mass	16.09 kg
Mass density	7850 kg/m^3^
Poisson's ratio	0.28
Elasticity modulus	200 GPa
Bending stiffness	20 Nm^2^
Length of each floor	34.3 cm

**Table 5.** Accuracy comparison between the harmonic motion and shear building tests.

**Testing**	**Frequency (Hz)**	**Amplitude (mm)**	**Difference Measure**
Harmonic Motion (from Table 3)	2	2	3.4%
Shear Building	1.98	1.96	6.2%
	(lowest frequency)	(standard deviation)	
